# The role of the C-domain of bacteriophage T4 gene 32 protein in ssDNA binding and dsDNA helix-destabilization: Kinetic, single-molecule, and cross-linking studies

**DOI:** 10.1371/journal.pone.0194357

**Published:** 2018-04-10

**Authors:** Kiran Pant, Brian Anderson, Hendrik Perdana, Matthew A. Malinowski, Aye T. Win, Christopher Pabst, Mark C. Williams, Richard L. Karpel

**Affiliations:** 1 Department of Physics, Northeastern University, Dana Research Center, Boston, Massachusetts, United States of America; 2 Department of Chemistry and Biochemistry, University of Maryland Baltimore County, Hilltop Circle, Baltimore, Maryland, United States of America; 3 Center for Interdisciplinary Research on Complex Systems, Northeastern University, Dana Research Center, Boston, Massachusetts, United States of America; Weizmann Institute of Science, ISRAEL

## Abstract

The model single-stranded DNA binding protein of bacteriophage T4, gene 32 protein (gp32) has well-established roles in DNA replication, recombination, and repair. gp32 is a single-chain polypeptide consisting of three domains. Based on thermodynamics and kinetics measurements, we have proposed that gp32 can undergo a conformational change where the acidic C-terminal domain binds internally to or near the single-stranded (ss) DNA binding surface in the core (central) domain, blocking ssDNA interaction. To test this model, we have employed a variety of experimental approaches and gp32 variants to characterize this conformational change. Utilizing stopped-flow methods, the association kinetics of wild type and truncated forms of gp32 with ssDNA were measured. When the C-domain is present, the log-log plot of *k vs*. [NaCl] shows a positive slope, whereas when it is absent (*I protein), there is little rate change with salt concentration, as expected for this model.A gp32 variant lacking residues 292–296 within the C-domain, ΔPR201, displays kinetic properties intermediate between gp32 and *I. The single molecule force-induced DNA helix-destabilizing activitiesas well as the single- and double-stranded DNA affinities of ΔPR201 and gp32 truncated at residue 295 also fall between full-length protein and *I. Finally, chemical cross-linking of recombinant C-domain and gp32 lacking both N- and C-terminal domains is inhibited by increasing concentrations of a short single-stranded oligonucleotide, and the salt dependence of cross-linking mirrors that expected for the model. Taken together, these results provide the first evidence in support of this model that have been obtained through structural probes.

## Introduction

Bacteriophage T4 gene 32 protein is a model single-stranded DNA binding protein (SSB), with well-established roles in DNA replication, recombination, and repair, as well as in the control of its own expression at the translational level.[1–7] There is a large body of work on the binding properties of 32 protein with single-stranded (ss) nucleic acids, as well as on the details of the protein’s domain structure.[4, 5, 8–17] Interaction with ssDNA or RNA is highly cooperative, involving protein-protein contacts between the N-domain (residues 1–21) of a nucleic acid-bound protein monomer and the core domain (residues 22–253, known as *III) of the adjacent bound protein.[4, 5] The core domain binds ssDNA in a positively-charged trough created by an OB-fold,[10, 18] a structural motif shared by other SSBs.[19, 20] The acidic C-domain (residues 254–301) is involved, directly or indirectly, in interactions with other T4 replication, repair, and recombination proteins.[14]

The C-domain also creates a “kinetic block” to the protein’s thermodynamically-predicted ability to lower the thermal melting temperature (Tm) of natural double-stranded DNAs.[4, 8, 9, 21, 22] The temperature range of these experiments was necessarily limited by the temperature at which the protein denatures (50–55°C). However, single molecule DNA stretching experiments, where the mechanically-induced helix→coil transition is observed at temperatures well below the protein denaturation point,[23–27] have provided new insights into the nature of the kinetic barrier.[28–32] These studies indicate that the critical factor governing the helix-destabilizing activity is the magnitude of the protein’s (non-cooperative) affinity for *double*-strandedDNA, which is significantly higher for the *I truncate (protein minus the C-domain) relative to intact protein.

**Fig 1:**
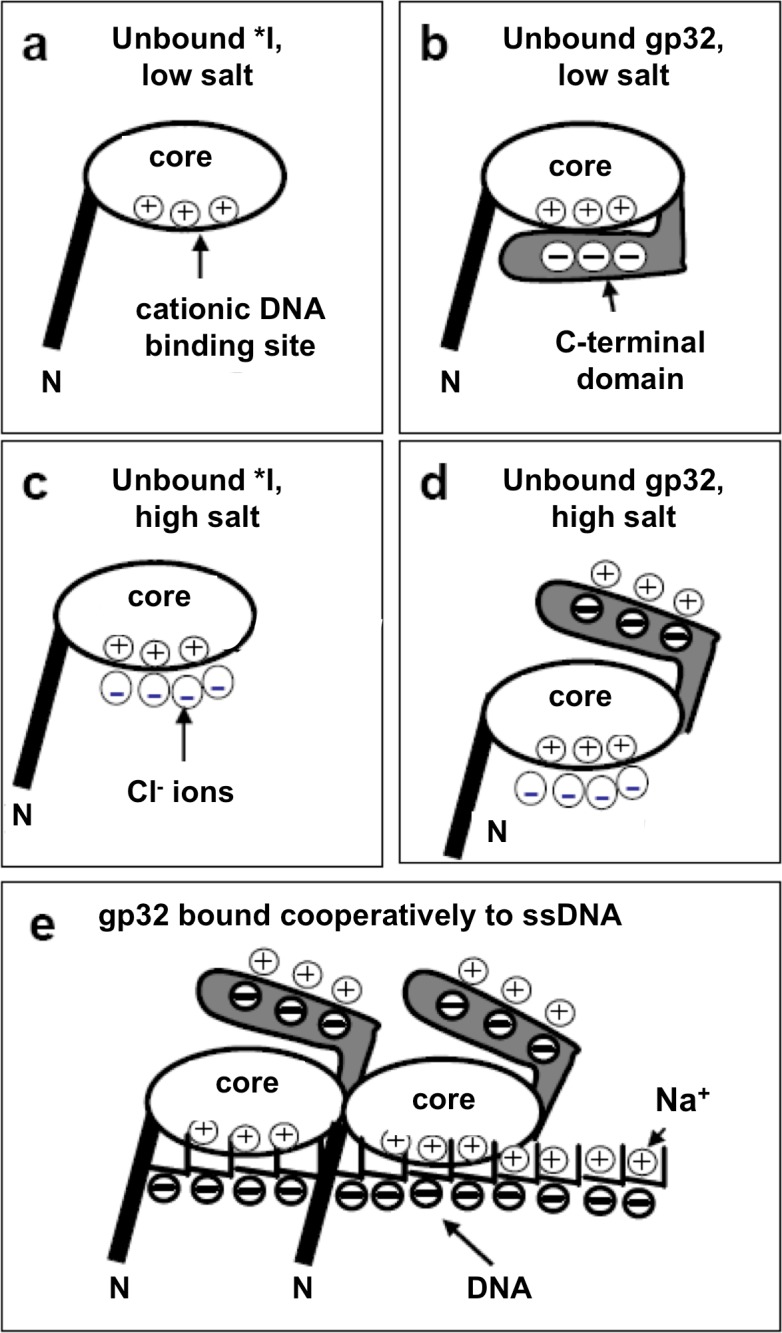
Model of electrostatic regulation of ssDNA binding to intact gene 32 protein (gp32) and protein lacking C-terminal domain (*I). a) In low salt, the DNA binding site of *I is always available for binding to DNA. b) In low salt, the gp32 C-domain can bind the cationic DNA binding site, thus blocking binding to DNA. c) In high salt, 4 Cl^-^ ions are condensed onto the cationic binding site of *I. d) In high salt, the C-domain of gp32 is unbound from the core, so gp32 resembles *I, with 4 Cl^-^ ions bound to the cationic DNA binding site on the core. e) When full length gp32 is bound to DNA, the gp32 C-domain is exposed to solution. Three Na^+^ ions are condensed onto the displaced C-domain. (*adapted from ref*.*32*).

DNA stretching experiments conducted over a wide range of salt levels were interpreted in terms of a model where, below 0.2 M Na^+^, the acidic C-domain of intact protein blocks the ssDNA binding surface on the core domain, and must dissociate from this site prior to binding nucleic acid (Fig 1).[31, 32] This “closed” ⇌ “open” conformational change, in pre-equilibrium with ssDNA binding, should be very salt dependent. When Na^+^ and Cl^-^ are the dominant ions, the conformational change is associated with counterion condensation of Na^+^ onto the C-domain and Cl^-^ onto the DNA binding site (Fig 1). In this regard, monoclonal antibodies that map within the C-domain and to the adjacent ~40 residues of core domain strongly cross-react with ssDNA.[14]

Additional evidence for the proposed conformational change is provided by a consideration of the kinetics of 32 protein association with single-stranded DNA. Lohman and Kowalcykowski published a detailed study on the binding kinetics of full-length 32 protein with single-stranded polynucleotides, using the stopped-flow method.[33] They interpreted their results in terms of a three step mechanism involving formation of (1) non-cooperative (monomeric protein) complexes via three-dimensional diffusion; (2) sliding (one-dimensional diffusion) of bound monomeric proteins to form contiguous clusters; and (3) equilibrium distribution into long clusters of cooperatively-bound protein. At moderate ionic strength (≤ 0.1 M NaCl), “strong” binding conditions, the initial non-cooperative binding step was seen to be rate determining. However, at high salt levels (≥ 0.2 M NaCl), where the affinity is significantly lower (“weak” binding), non-cooperative binding is in pre-equilibrium to the rate-determining formation of contiguous clusters by 1D diffusion, the second step in the process.

The measured salt dependence of the association rate constant reflected these two binding modes: At high salt concentration, weak binding conditions, the log *k*-log [NaCl] plot had a negative slope (~ -6), similar to the salt dependence of the affinity of the protein for an isolated single-stranded site (*K*_*ss*_), log *K*_*ss*_ *vs*. log [NaCl] (the subsequent protein-protein association steps are salt-independent). This reflects the release of Na^+^ ions from the nucleic acid and Cl^-^ ions from the protein upon formation of the complex. However, a striking discontinuity in this plot was seen at low [NaCl]: between 0.02 and 0.1 M salt, the log *k*—log [NaCl] plot showed a distinctly *positive* slope (~ 3). This highly unusual salt dependence was interpreted as the result of electrostatic repulsion between the negatively-charged polynucleotide and the negatively-charged protein (the pI of 32 protein is about 5)[33], a factor that becomes more significant with the decreased charge shielding at lower ionic strength. A detailed re-examination of the salt dependencies of single molecule DNA stretching data and previous bulk DNA binding results showed that the kinetic results could be satisfyingly explained by our proposed conformational change.[31, 32, 34, 35] Below 0.2 M NaCl, the protein is initially in the closed form. Upon formation of the open form, the release of the anionic C-domain from its cationic surface on the core results in the binding of ~3 Na^+^ ions from the bulk solution to the now-exposed C-domain, and a positive slope in the log *k vs*. log [NaCl] plot.[32]). The [Cl^-^] is too low to affect binding of these anions to the uncovered core surface, but at higher [NaCl], above 0.2 M, where Cl^-^ ions are bound to this cationic surface, the rate-determining step follows the *release* of both these Cl^-^ ions as well as Na^+^ (from DNA) to the bulk solution, and the slope of the log *k*–log [NaCl] plot is therefore negative. Since at this [NaCl] the protein is already in its open form prior to interacting with the ssDNA, there is no binding or release of Na^+^ ions at the C-domain.

Clearly, the proposed salt-dependent conformational change is a critical factor in the functioning of gene 32 protein. Although there is substantial thermodynamic evidence in favor of this model, all of it is indirect. Here we provide strong evidence for the conformational change, utilizing stopped-flow kinetic, DNA stretching, and cross-linking methods. These experiments employ gene 32 protein variants mutated within the C-domain (gene 32 protein from the T4 mutant strain ΔPR201, which lacks residues 292–296, and a truncation extending to residue 295 [36]), as well as the canonical truncated forms of the protein (*I: protein minus C-domain; *II: protein minus N-domain; *III: protein minus both N- and C-domains, “core domain”). With this wide variety of methods and materials, including direct tests through mutations and cross-linking, our results clearly support a regulatory function for the C-domain, controlling the binding of ssDNA as it interacts with and is released from its binding site on the core domain.

## Materials and methods

### Proteins and nucleic acids

Full length gene 32 protein and its truncated forms were prepared as previously described.[9, 10]For preparation of the ΔPR201 variant, the region of the gene to be altered was removed from the gp32 expression vector pYS6 (7436 bp) by digestion with BamHI and SacI and inserted into the multiple cloning site of pUC19. pUC19, a smaller vector (~2600 bp), was utilized in order to facilitate site-directed mutagenesis. The deletion mutations were introduced into the fragment of gene 32 in pUC19 using QuikChange site-directed mutagenesis (Stratagene). The primers used flanked the sites to be removed (5’-AGCTCTGGTAGTTCATCTAGTGCTGATGACCTTTTGAATGACCTT-3’, forward, and 5’-AAGGTCATTCAAAAGGTCATCAGCACTAGATGAACTACCAGAGCT-3’, reverse). The altered plasmids obtained were transformed into *E*. *coli* DH5α. Colonies containing the deletions were identified by restriction analysis, and subsequently verified by sequencing. The appropriate fragments were then inserted into digested pYS6. Induction, overexpression, and purification of the mutated protein was performed as described for wild-type and truncated proteins.[9, 10] The 1–295 truncation mutant was produced via the Quikchange method, verified by DNA sequencing, and overexpressed and purified in the same manner. Poly(deoxythymidylic acid) was obtained from Sigma.

The C-terminal domain (CTD) of gene 32 protein was prepared by a modification of the procedure of Hurley *et al*.[36] BL21 *E*. *coli* cells, transformed with pMM1 plasmid (gift of Scott Morrical, University of Vermont), were induced in mid-log phase with 2 mM IPTG. Cell extracts, in 20 mM Tris-HCl, pH 8.0, 150 mM NaCl, 1 mM EDTA, 1 mM β-mercaptoethanol, 10 mM benzamidine-HCl, 0.5 mM PMSF, 20% (v/v) glycerol, were treated with 2 mg/mL protamine, and ultracentrifuged at 100,000 g for 30 min. The clarified extract was diluted, so as to reduce the [NaCl], and applied to a Q Sepharose Fast Flow anion exchange column, equilibrated in the above buffer with 0.1 M NaCl. The column was subjected to a salt gradient, and the CTD was seen (via SDS-PAGE) to elute between 0.25 and 0.30 M NaCl. Fractions containing the CTD were concentrated and further purified via successive runs on Centriprep 30 and Centricon Ultra membranes (30 kDa MWCO, Amicon), which, as observed on SDS-PAGE, eliminated all contaminating polypeptides with molecular weights higher than that of the CTD. The flow-through from the 30 kDa membranes was then applied to Ultra-4s membranes (5 kDa MWCO), and the retentate yielded >95% pure CTD. CTD concentration was determined spectrophotometrically, based on its content of six phenylalanine residues, ε_258_ = (6 x 190) M^-1^cm^-1^.[37]

### Stopped-flow experiments

A Hi-Tech KinetAsyst stopped-flow instrument was used to collect kinetic data. Generally, 50 μL of protein solution was mixed with an equal volume of nucleic acid, and each run averaged at least 10 mixings, typically 15–25; each data point has an uncertainty of ±25%. The KinetAsyst software provided with the instrument was used to fit the average of the decays for each run. The software calculatesthe Standard Error of the Estimate for the kinetic parameters. The temperature of the reactants was maintained at 25°C, with the exception of the temperature dependence experiments. The reaction was monitored by the quenching of protein tryptophan fluorescence. Unless otherwise stated, stopped-flow experiments were performed in 10 mM Tris-HCl, pH 8.3, 0.1 mM Na_2_EDTA (“buffer T” of Lohman and Kowalczkowski[33]) and the indicated concentration of NaCl.

### Single-molecule DNA stretching

A dual-beam optical tweezers instrument, previously described[38], was used to capture two 5.6 μm-diameter polystyrene spheres (Bangs Labs, Fisher, IN), one on the end of a glass micropipette and one in the optical trap. A single bacteriophage-λ DNA molecule, labeled with biotin on its 3’ ends, was captured between the beads. The DNA molecule was then stretched by moving the micropipette in steps ranging from 5 nm to 250 nm at 1 step/s. After verifying that the expected DNA force-extension curve was obtained, a solution containing a fixed protein concentration was exchanged with the buffer surrounding the DNA molecule by flowing four to five cell volumes of liquid past the captured molecule.

### Cross-linking

Cross-linking of the C-terminal domain to the core domain was achieved by the modification of the two-step method of Grabarek and Gergely.[39] 20 μM CTD and 4 μM core domain (*III), in 40 mM MES, pH 6.0, 5% glycerol, were mixed with 1.25 mM N-hydroxysuccinimide (NHS) and 0.5 mM1-Ethyl-3-[3-dimethylaminopropyl] carbodiimidehydrochloride (EDC, Pierce) at room temperature. After 20 min, β-mercaptoethanol was added to 5 mM, and cross-linking proceeded for 20 min, after which NH_2_OH·HCl was added to 5 mM to quench the reaction. Coomassie brilliant blue R-250 used in SDS-PAGE experiments was purified by the method of Kundu *et al*.[40]

## Results

### Cooperatively-binding proteins characteristically display two relaxation times

**Fig 2:**
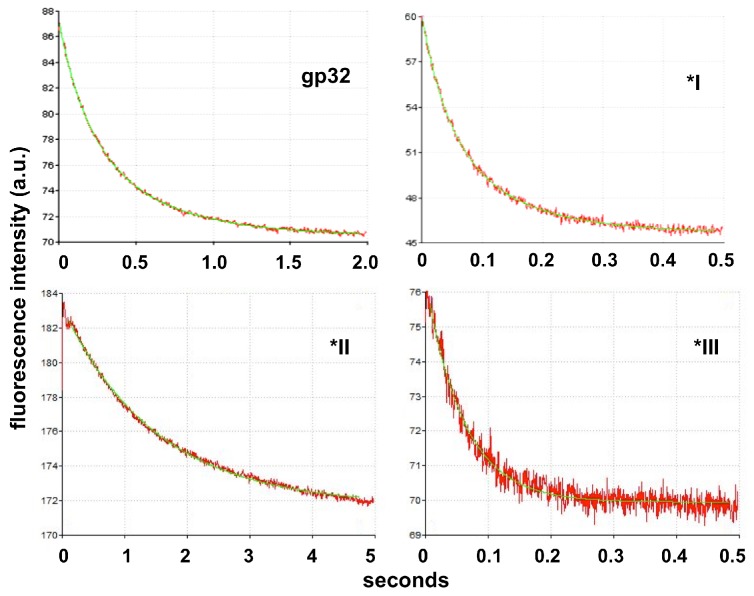
Representative kinetic traces of poly(dT) mixed with full-length (gp32) and truncated forms of gene 32 protein. 0.125 μM protein (gp32 and *I) or 0.063 μM protein (*II and *III) + 2.6 μM(p) (residue concentration) poly(dT) in buffer T (10 mM Tris-HCl, pH 8.3, 0.1 mM Na_2_EDTA) at 25°C.The gp32 and *I data were fit by a biexponential decays, the *II and *III data as single exponential decays. The fit is seen as green line within the (red) data. First order rate constants, *k* (*τ*^*-1*^) for these traces are as follow: gp32: *k*_*fast*_ = 5.3 s^-1^, *k*_*slow*_ = 1.8 s^-1^; *I: *k*_*fast*_ = 24 s^-1^, *k*_*slow*_ = 8.5 s^-1^; *II: *k* = 0.67 s^-1^; *III: *k* = 19 s^-1^. For *I, the Standard Error of the Estimate (SEE) was 0.23% for both rate constants; for *II and *III, the SEE for *k* was, respectively, 0.50% and 1.3%.The amplitude of the slow decay for the *I data is 20% of the total fluorescence change, and the SEE for the fast decay and slow decay amplitudes were, respectively, 0.50% and 1.3%.

Lohman and Kowalczykowski found that the association kinetics of full-length gene 32 protein with single-stranded DNAs were characterized by two first-order decays.[33]Preliminary experiments [33] indicated that the *I truncate also has this property, and we observed biexponential decays with both full-length and *I protein. Typical decays, obtained at 0.02 M NaCl, are shown in Fig 2. Both these proteins bind cooperatively to single-stranded nucleic acids, whereas the association kinetics of *II and *III, which both bind non-cooperatively, are well-fit by a single exponential decay (Fig 2). Lohman and Kowalczykowski suggested that the slow decay is likely related to the occurrence of binding cooperativity (see ref. 33 for a detailed discussion). As was observed for the full-length protein,[33] the amplitudes of the slow decays seen with *I were smaller than those of the fast decays. The slow decay amplitudes were usually less than 25% of the total time-dependent change in fluorescence; there was no obvious pattern in the variation of the slow decay amplitudes with experimental conditions (protein and DNA concentrations, [NaCl], temperature).[33] As we will show, the time constants for *II and *III are similar to the fast decay times of whole protein and *I, respectively. This indicates, as Lohman and Kowalczykowski previously suggested,[33] that the fast decay is associated with the diffusion-controlled binding of protein to the nucleic acid.

### Association kinetics are determined under pseudo-first order conditions

**Fig 3:**
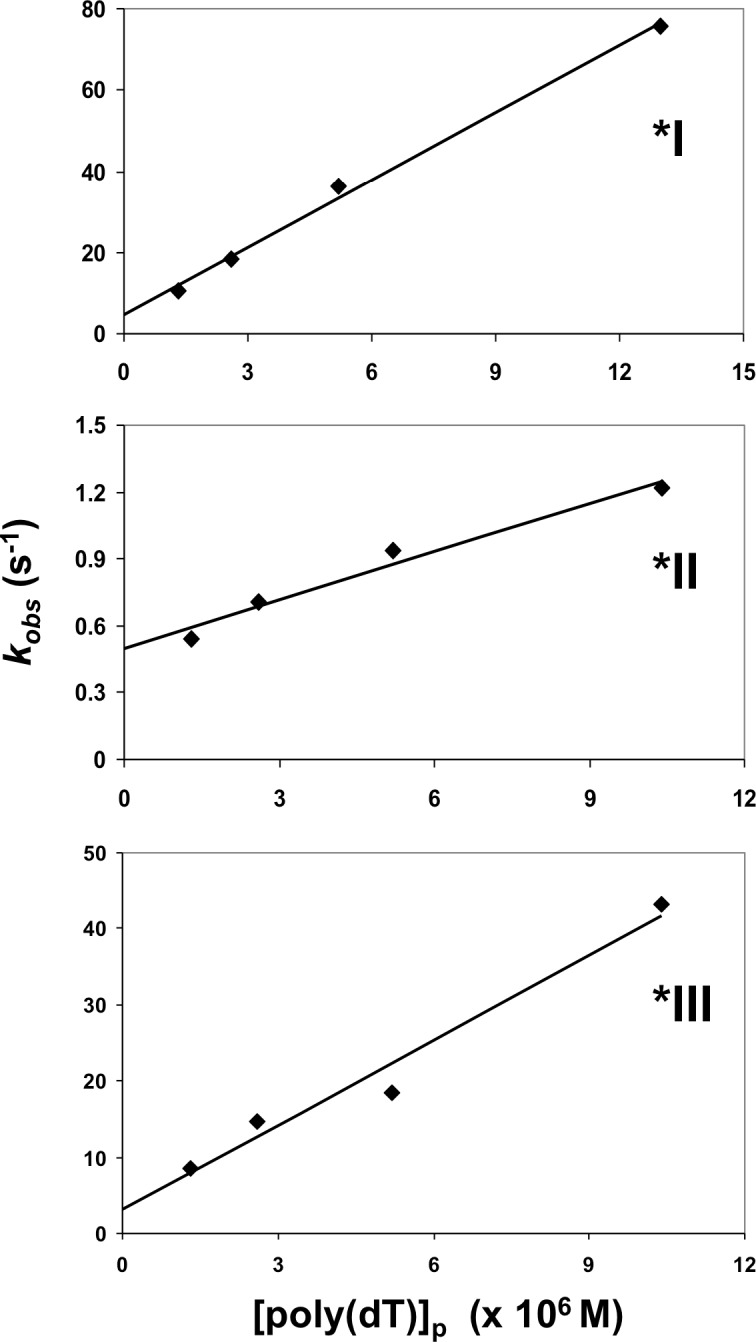
Linear dependence of association kinetics of *I, *II, and *III on poly(dT) concentration. Conditions as in Fig 2, other than the (indicated) poly(dT) residue concentration. The slopes, intercepts, and coefficients of determination (*R*^*2*^) for each of the plots is as follows: *I: 5.5 x 10^6^ M^-1^s^-1^, 4.6 s^-1^, 0.995; *II: 7.2 x 10^4^ M^-1^s^-1^, 0.50 s^-1^, 0.969;*III: 3.7 x 10^6^ M^-1^s^-1^, 3.1 s^-1^, 0.968.With a zero intercept, the values for *I and *III are, respectively, 6.0 x 10^6^ M^-1^s^-1^, *R*^*2*^ = .981 and 4.1 x 10^6^ M^-1^s^-1^, *R*^*2*^ = .949.

The bimolecular association of protein (P) and DNA (D) to form complex (PD), and the dissociation of the complex, can be simply described as

$$P+D\overset{k_{f}}{\underset{k_{r}}{⇄}}PD$$ (1)

where *k*_*f*_ and *k*_*r*_ are the second order association and dissociation rate constants, respectively. Under conditions where one of the reactants is present in great excess relative to the other (in this case, [D] >> [P]), the approach to equilibrium follows the following relationship:

$$\tau^{-1}=k_{f}\left[D\right]+k_{r}$$ (2)

where τ is the relaxation time of a first-order decay (τ^-1^ = *k*_*obs*_). Under these pseudo-first order conditions, plots of τ^-1^*vs*. [DNA] should be linear, with the slopes yielding the second-order association constant, *k*_*f*_, and the y-intercepts providing an estimate of the dissociation constant, *k*_*r*_. This relationship clearly holds for the fast decay of *I, as well as the single exponential decays of *II and *III (Fig 3). The results with the slow exponential decays observed for *I–poly(dT) association, which as we have noted generally represented a small portion of the total decay amplitude, were less consistent. In general, the slow rate increased with increasing [poly(dT)], but with the small amplitudes and variation from experiment to experiment (not seen with the fast decays), we chose not to quantitatively analyze the slow decays.

As seen in Fig 3. for a given poly(dT) concentration at 0.02 M NaCl (strong binding conditions), the *k*_*obs*_ (*τ*^*-1*^)for *I and *III are similar. The corresponding relaxation times for *II are significantly longer, and similar to the fast decays of full-length gp32.[33]

The y-intercepts for the *k*_*obs*_ *vs*. [poly(dT)] plots in Fig 3 for *I and *III are approximately 0, so that *k*_*obs*_ ≈ *k*_*f*_[D] for these two truncates. The slopes obtained for the corresponding plots with the intercepts set at 0 vary by only about 10% (see Fig 3 caption for details). The plot for *II clearly does not have a y-intercept of 0, and *k*_*f*_ values for this truncate are therefore approximate. Overall, the results are consistent with the existence of a closed conformation (in gp32 and *II) at these low salt conditions, where the need to undergo a conformational change involving the blocking C-domain slows down the association rates. This impediment does not exist for *I and *III, which lack the C-domain.

### The presence of the C-domain has a striking effect on the salt dependence of the association rate

**Fig 4:**
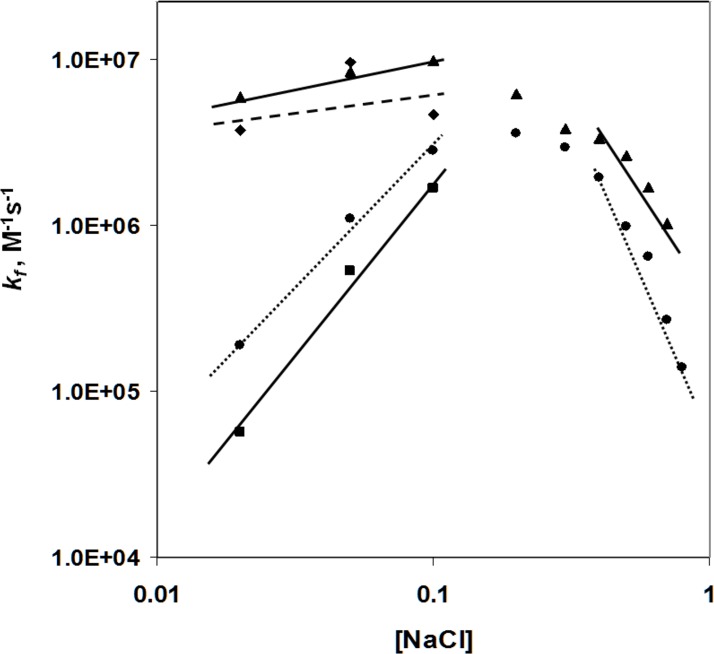
Salt dependence of *k*_*f*_ (second-order association rate constant). Conditions as in Fig 2, other than the (indicated) [NaCl]. *k*_*f*_ for *II is an approximation (see text). ▲, *I; ■, *II; ♦, *II; ●, full-length 32 protein (gp32), from ref. 33.

The effect of salt on the fast association rate constant for *I was determined over a wide range of NaCl concentrations (0.02–0.7 M, Fig 4). For comparison, the previous results of Lohman and Kowalczykowski[33] with full-length 32 protein are also shown. As was the case for intact 32 protein, the log *k*_*f*_ *vs*. log [NaCl] plot for *I shows a striking discontinuity between 0.1 and 0.4 M NaCl. At salt levels where the rate determining step is governed by the formation of contiguous protein clusters (> 0.3 M NaCl), the slope of the log *k*_*f*_ *vs*. log [NaCl] (~ -3) for *I is similar to that of the intact protein; the rate constants for *I are about three times greater than for the intact protein. Between 0.02 and 0.1 M NaCl, where the rate determining step is the formation of non-cooperatively bound protein (strong binding conditions), the dependence of the association rate constant on [NaCl] is vastly different for the two proteins. With *I,*k*_*f*_ increases moderately with increasing salt concentration; the log *k*_*f*_ *vs*. log [NaCl] plot has a slope of about 0.3. The corresponding slope for intact protein is about five times this value (1.7). Although the magnitude of *k*_*f*_ was always larger for *I, the differences were seen to be greatest at 0.02 M NaCl, where the forward rate was thirty-fold greater than for 32 protein.

The association kinetics of the two forms of the protein that lack the N-domain, *II (core and C-domains) and *III (core domain), were also studied. Since neither of these truncates bind ssDNA cooperatively, we restricted these experiments to “strong” binding conditions, 0.02–0.1 M NaCl. As can be seen in Fig 4, the slopes of the log *k*_*f*_ *vs*. log [NaCl] for *II (2.1) and *III (0.2) are very similar to those of intact 32 protein and *I, respectively. Although the data for *II is an approximation, since the dependence of *k*_*obs*_ *vs*. [poly(dT)]_p_ for this truncate does not yield a y-intercept of 0, the similarity of the [NaCl] dependence of *II and full-length protein is remarkable.

These results indicate that, under strong binding conditions, the presence of the C-domain in the protein brings about a significant positive slope in the salt dependence, and when it is absent, there is little variation of association rate with salt. Taken together with the corresponding data obtained with intact protein and *I, it is clear that the C-domain has a critical effect on the salt dependence below 0.2 M NaCl. The results are fully consistent with the uptake of Na^+^ ions by the C-domain as a consequence of the closed → open conformational change that occurs under these conditions.

### The variation of association kinetics with temperature is dependent on the presence of the C-domain

**Fig 5:**
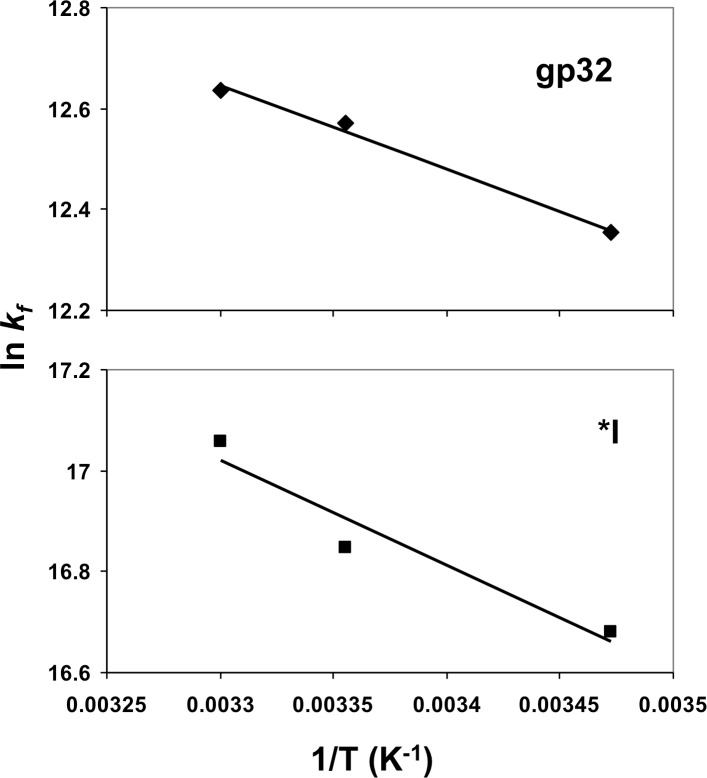
Temperature dependence of *k*_*f*_ for full-length 32 protein (gp32) and *I. Conditions as in Fig 2.

We measured the variation of *k*_*f*_ between 15 and 30°C in 0.02 M NaCl, the salt concentration where the association rates among the different forms of the protein are most different; the results are tabulated in Table 1. For both whole protein and *I, there is only a very small effect of temperature on rate. In both cases, ln *k*_*f*_ *vs*. 1/T plots (Fig 5) yielded an activation enthalpy, ΔH^‡^, of about 15 kJ/mol, consistent with a diffusion-controlled reaction. In contrast, the *entropy* of activation, ΔS^‡^, is significantly more negative for whole protein (~-100 kJ/mol) than for *I (~-45 kJ/mol). Thus, the much lower values of *k*_*f*_ for whole protein relative to *I are due to the significantly more negative value of ΔS^‡^. The analogous data for the non-cooperatively-binding *II and *III (both lacking the N-domain, *III also lacking the C-domain) shows the same trend. There is relatively little variation of rate with temperature (ΔH^‡^ ~ 25 kJ/mol), and the ΔS^‡^ values for *II and *III are, respectively, ~-60 kJ/mol and ~-34 kJ/mol. The negative entropies of activation, seen for all the proteins, indicate that the transition states are more ordered than the reactants. The effect is accentuated when the protein possesses the C-domain. Although the values for ΔS^‡^, obtained by extrapolation, are approximate (and the kinetic data for *II is subject to the caveat discussed in the previous section), there clearly are significant differences in the activation entropies, ΔΔS^‡^ ~ -50 JK^-1^mol^-1^ for whole protein vs. *I, and ~ -25 JK^-1^mol^-1^ for *II vs. *III. We further discuss this observation in the Discussion.

**Table 1:** Activation parameters derived from the temperature dependence of intact and truncated protein-ssDNA association kinetics.

Protein	ΔH^‡^, kJ mol^-1^	ΔS^‡^, J K^-1^ mol^-1^	ΔG^‡^, kJ mol^-1^ at 25.0°C
gp32	14±4	-100±20	45±4
*I	17±4	-45±10	31±4
*II	28±5	-60±10	45±4
*III	24±5	-34±10	36±4

### Modification of the C-terminal domain alters binding kinetics

**Fig 6:**
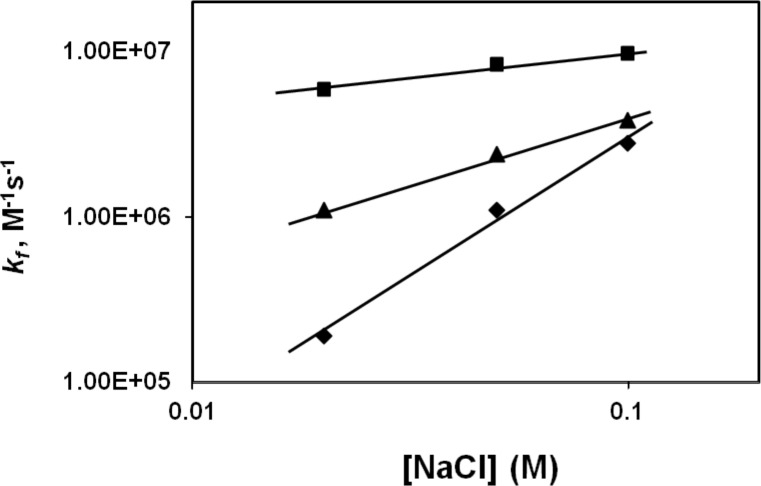
Comparison of ΔPR201-poly(dT) association kinetics with that of full length and *I protein. Conditions as in Fig 2.

Additional kinetic evidence for the conformational change involving the C-domain was provided by stopped-flow association kinetics utilizing the ΔPR201 variant of gene 32 protein, where an in-frame 15-nucleotide deletion results in the loss of residues 292–296 (Thr-Asp-Leu-Asp-Asp), and, consequently, three negative charges.[36] Interestingly, the salt dependence of the association kinetics of this protein with poly(dT) under strong binding conditions showed a slope in between that of full-length and *I proteins (Fig 6). This result suggests that the modified C-domain of the ΔPR201 variant has a reduced, but not totally absent, ability to form the “closed” conformation.

### C-domain mutants display helix-destabilizing activity

**Fig 7:**
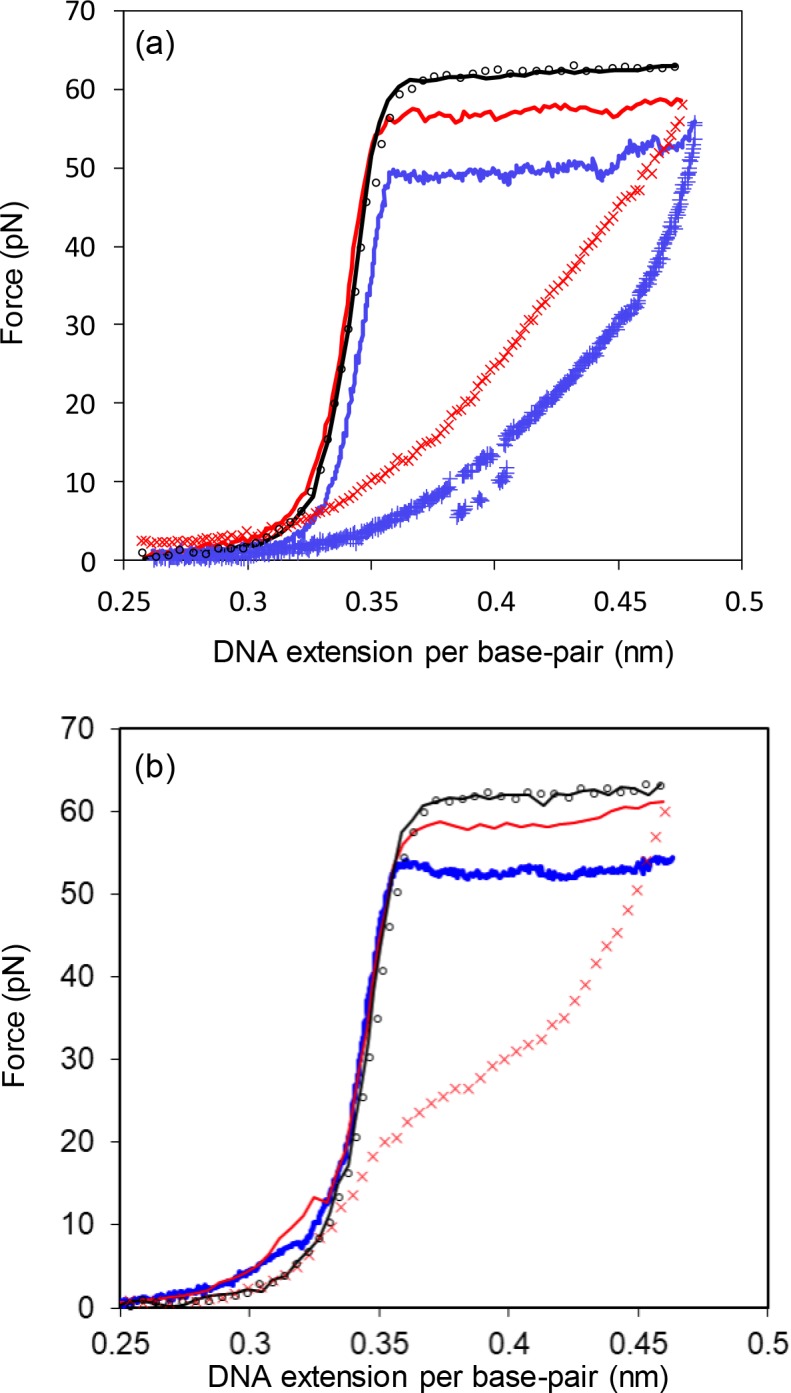
Typical DNA stretching and relaxation curves for ΔPR201 and 1–295. (a) Stretching (solid line) and relaxation (symbols) curves for λ-DNA in 10 mM Hepes pH 7.5, 0.1 M [Na^+^] (95 mM NaCl and 5 mM NaOH) in the absence of protein (black) at pulling rate 250 nm/s and in the presence of 200 nM 1–295 at the pulling rate 250 nm/s (red) and 400 nM 1–295 at 25 nm/s (blue). (b) Stretching (solid line) and relaxation (symbols) curves for λ-DNA in 10 mM Hepes pH 7.5, 0.1 M [Na^+^] in the absence of protein (black) at pulling rate 250 nm/s and in the presence of 200 nM ΔPR201 at the pulling rate 100 nm/s (red) and 25 nm/s (blue). Due to breaking of the DNA at the end of the transition, we do not show a relaxation curve for 25 nm/s in this case.

Having shown that a deletion of five residues from the C-domain would alter the salt dependence of the ssDNA association kinetics of gene 32 protein, it was of interest to determine if other properties of the protein were also affected. Accordingly, using single-molecule DNA stretching methods, we measured the DNA helix-destabilizing activity of ΔPR201 and “1–295”, a truncated protein whose C-terminus (Asp-295) is located within the ΔPR201 deletion sequence. Interpretation of the stretching results provides equilibrium constants for the association of the protein with ss and dsDNA, as well as the rate of protein finding its binding site on ssDNA and the binding site size.[28, 30, 31] We have previously collected and interpreted stretching data for full-length protein (gp32) and the *I, *II, and *III (core domain) truncates. Although full-length protein and core domain can be shown to possess some helix-destabilizing activity at moderate (0.1 M) Na^+^, over a short (~1 min) time scale neither of these proteins showed any effect on the force needed to denature single λ-DNA molecules, whereas*I reduced the this force, the overstretching force, by about 25 pN under.[28] Typical DNA stretching and relaxation curves for ΔPR201 and 1–295 at these conditions are shown in Fig 7. Both proteins reduce the overstretching force, but by considerably less than does *I. The cooperativity of the stretching transition unaffected by the presence of protein, occurring over a very narrow force interval of ~3 pN. The intermediate helix-destabilizing properties of these two mutant proteins mirror the stopped-flow results seen with ΔPR201 and further support a role for the C-domain in regulating this activity of the protein.

### Mutations in the C-domain alter the rate dependence of the DNA unwinding force

**Fig 8:**
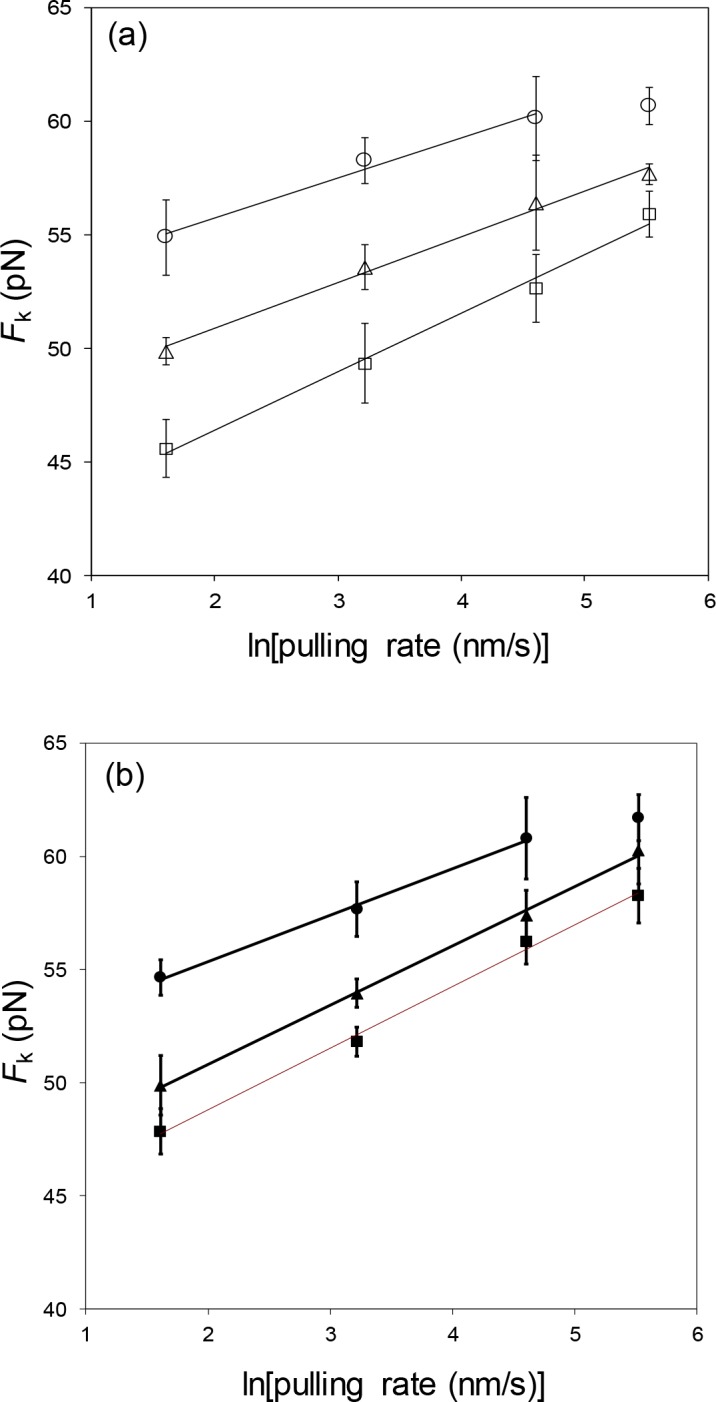
Dependence of DNA overstretching force, *F*_*k*_, on the natural logarithm of pulling rate. Data are shown as symbols and linear fits as solid lines. (a) Measurements in the presence of 1–295 at the following concentrations: 100 nM (open circle), 200 nM (open triangle), 400 nM (open square). (b) Measurements in the presence of ΔPR201: 100 nM (filled circle), 200 nM (filled triangle), and 400 nM (filled square). The [Na^+^] was 0.1 M in all cases.

The strong hysteresis observed in the stretching and relaxation curves seen in Fig 7 is similar to that observed for full length protein and *I.[28] The system does not achieve equilibrium at the rates of stretching and relaxation utilized, 25 and 250 nm/s for 1-295.Although we were unable to observe a relaxation curve for 25 nm/s in the presence of ΔPR201, we expect that it would likely also show significant hysteresis, as DNA breaking was typically observed after DNA became fully single-stranded at the end of the overstretching transition in this case. As was observed with gp32 and *I, the non-equilibrium overstretching force varies with pulling rate, whereas in the absence of protein, the system is almost completely reversible. The non-equilibrium data can be used to calculate kinetic and equilibrium parameters for the protein-DNA interaction, as follows:

We have shown that the nonequilibrium DNA unwinding force (*F*_*k*_) is a function of pulling rate (ν), expressed as

$$F_{k}\left(\nu \right)=\frac{k_{B}T}{n\Delta x}\cdot \ln\left(\frac{\nu }{2n\Delta xk_{a}}\right)+F_{m}^{nc},$$ (3)

where F_m_^nc^ is the equilibrium DNA melting force associated with noncooperative binding to ssDNA, and is close to the value of *F*_*m*_^*0*^, the equilibrium DNA melting force in the absence of protein. *k*_*a*_ is the rate of a single protein finding its contiguous binding site, which is created by the melting of *n* base pairs at the boundary between dsDNA and protein-covered ssDNA at the ends of the DNA molecule.[30, 34, 35] The extension per base pair, Δ*x*, upon dsDNA melting with concurrent protein ssDNA binding is determined directly from our stretching curves. The data for both 1–295 and ΔPR201 follow the predicted linear dependence of *F*_*k*_ on *ln*(ν) (Fig 8). The slopes of analogous plots for gp32 and *I yielded the number of nucleotides of ssDNA that bind one protein molecule, *n* = 7±1, in accord with previous studies.[11, 33, 41] Using this value for binding site size, *k*_*a*_ can be calculated by extrapolation of the plots in Fig 8 to *ln(ν/2nΔx*^.^*k*_*a*_*)* = 0, where *F*_*k*_ = *F*^*nc*^_*m*_. At 200 nM protein concentrations in 0.1 M Na^+^, we obtain for 1–295 and ΔPR201, respectively, *k*_*a*_ *=* 1.8±0.5 x10^2^ s^-1^ and 1.1±0.3 x10^2^ s^-1^, essentially equal within experimental error. The magnitudes of these rate constants fall in between the analogous values at the same conditions for full-length and *I protein, 31±3 and 2.8±0.8 x10^4^ s^-1^.[30]

In a previous DNA stretching study on gp32 and *I, we showed that *k*_*a*_ exceeds the 3D diffusion limit and varies quadratically with protein concentration.[30] The dominant mechanism for the protein locating its single-stranded DNA binding site is one-dimensional sliding on double-stranded DNA.[30, 34] Assuming that the protein finds its ssDNA binding site before dissociating from the DNA molecule,[30]

$$k_{a}={\left(2\Theta /n_{ds}\right)}^{2}\cdot k_{s}$$ (4)

where *k*_*s*_ is the 1-D sliding rate of gp32 on dsDNA, θ is the probability of noncontiguous binding of the protein to dsDNA per nucleotide, and *n*_*ds*_ is the protein binding site size on dsDNA in nucleotides. Θ, which varies between 0 and 1, and can be calculated by use of the McGhee-von Hippel isotherm for non-cooperative binding, where steric occlusion of the binding site by a bound protein prevents simultaneous binding by another protein,[42]

Θ=Kds⋅n⋅C(1−Θ)n(1−Θ+Θn)n−1 (5)

*C* is protein concentration, *K*_*ds*_ = *k*_1_/*k*_−1_, and is the non-contiguous (non-cooperative) binding constant, and *k*_1_ is the non-contiguous association rate. We have used Eq (5) to calculate values of Θ, which in turn were used to fit our measured *k*_a_ (*C*) dependence *via* Eq (4).

**Fig 9:**
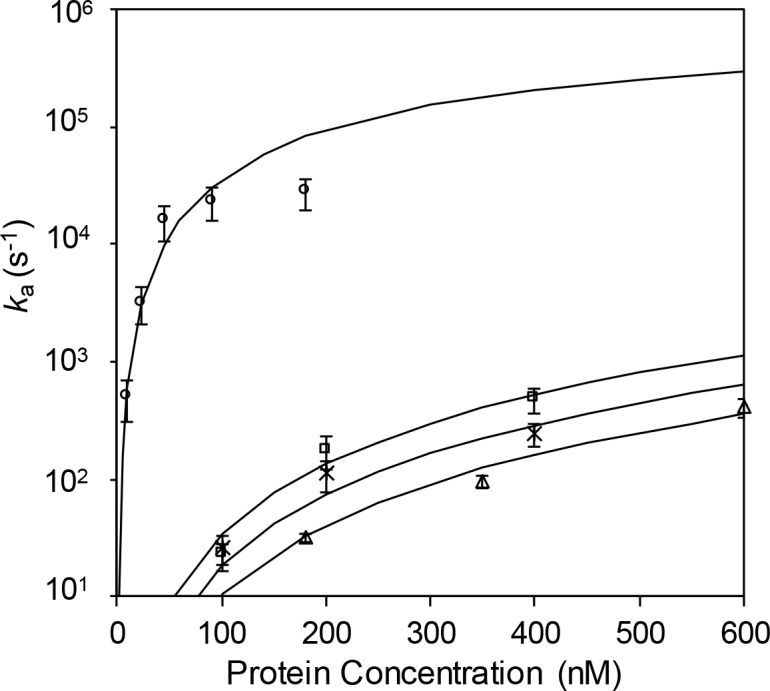
Protein concentration dependence of the association rate *k*_a_. The rate was determined for 1–295 (open square) and ΔPR201 (cross) from the data shown in Fig 8 by solving Eq (4) for *k*_a_ at each concentration. Lines are fits to the data using the McGhee and von Hippel isotherm, Eq (5). Protein concentration dependence of the association rate *k*_a_ for *I (open circle) and gp32 (open triangle).[30].

Fits of *k*_a_(*C*) for 1–295 and ΔPR201 for 0.1 M Na^+^ are shown in Fig 9. In this calculation, we use the same salt-independent parameters, *n*_ds_ = 7 and *k*_s_ = 10^7^ s^-1^, which were used for both *I and gp32.[30, 43] The only fitting parameter is the binding constant to dsDNA (*K*_*ds*_). The values of *K*_*ds*_ (tabulated in Table 2) are found to be 5.95±0.79 x 10^3^ and 4.39±0.57 x 10^3^ for 1–295 and ΔPR201, respectively. These values are 1.5 to 2 times *larger* than that of gp32 (3.00±1.00 x 10^3^) and about 40 to 60 times *smaller* than that of *I (2.53±0.5 x 10^5^ in 0.1 M Na^+^).[30] Thus, alterations within the C-domain increasethe affinity of the protein for double-stranded DNA, but not nearly as much as the removal of the entire domain, consistent with the conformational change model.

**Table 2:** Equilibrium parameters of gp32, *I, 1–295, and ΔPR201 binding to ssDNA and dsDNA in the range of 0.05 M to 0.2 M NaCl determined from single-molecule force-induced melting experiments.

Protein	*K*_ss_(M^-1^)	*K*_ds_(M^-1^)
gp32	3.36±0.30 x10^4^	3.00±1.00 x10^3^
*I	1.50±0.10 x10^6^	2.53±0.50 x10^5^
1–295	9.93±1.14 x10^4^	5.95±0.79 x10^3^
ΔPR201	1.02±0.14 x10^5^	4.39±0.57 x10^3^

Values are reported as mean ± standard error.

### C-domain mutant proteins show reduced affinities for ssDNA

The DNA stretching method can also be used to obtain equilibrium constants for the binding of proteins to single-stranded (*K*_*ss*_) DNA. Clearly, in the presence of 1–295 or ΔPR201, DNA melting is slow and doesn’t reach equilibrium under the stretching rates utilized. Ideally, at a slow enough pulling rate, the actual kinetic (non-equilibrium) unwinding force, *F*_*k*_, should decrease, and eventually reaching the rate-independent equilibrium melting force, *F*_*m*_. However, such extremely slow pulling rates are experimentally unrealistic, and we have utilized an alternative approach for measuring the equilibrium force in the presence of these proteins.[28, 31] The DNA is rapidly extended to ~0.42 nm/bp, about one third of the way into the overstretching plateau, i.e. where approximately one third of the DNA base pairs are melted. The force then exponentially decays to the equilibrium value, *F*_*m*_. The characteristic time of this force convergence is on the order of 1–30 minutes. In concert with the equilibrium nature of *F*_*m*_, the same force is obtained when the DNA molecule is first overstretched through the ds/ssDNA transition, then released quickly to the same end-to-end extension (*i*.*e*., 0.42 nm), and fixed in this position for a sufficiently long duration.[28, 31] *F*_*m*_ does not depend on the initial nonequilibrium conditions, such as the initial pulling rate and the corresponding kinetic unwinding force.[28]

We can relate the equilibrium melting force in the presence of the protein with this protein’s binding constant to ssDNA, *K*_ss_:[31]

$$F_{m}^{}\approx F_{m}^{0}-\frac{k_{B}T}{\Delta x}\cdot \frac{2}{n_{ss}}\cdot \ln\left(1+K_{ss}\omega C\right)$$ (6)

where $F_{m}^{0}$ and *F*_*m*_ are the equilibrium melting forces of DNA in the absence and presence of protein, respectively. *K*_*ss*_ is explicitly a function of the measured shift in equilibrium melting force, $\Delta F_{m}=F_{m}-F_{m}^{0}$:

$$K_{ss}=\frac{1}{\omega C}\cdot \left\{\exp\left(-\frac{n_{ss}\Delta x}{2k_{B}T}\cdot \Delta F_{m}\right)-1\right\}$$ (7)

**Fig 10:**
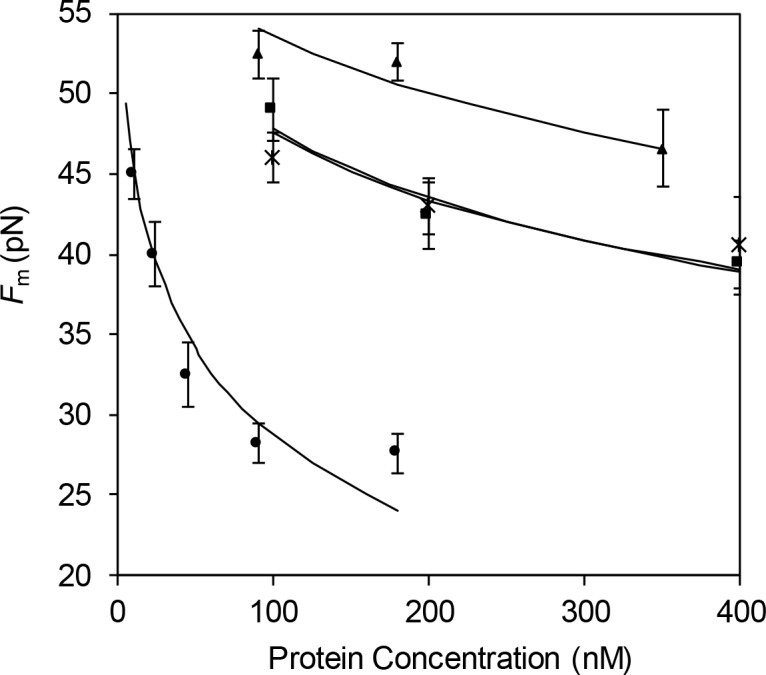
Dependence of equilibrium DNA melting force on protein concentration. Each symbol represents ≥ 3 experimental observations. Measurements are shown in the presence of 1–295 (black square), ΔPR201 (cross), gp32 (triangle) and *I (circle) in 100mM Na^+^. Lines are fit to data using Eq (6).

*K*_*ss*_ is calculated with the assumption that the ionic strength-independent values of the cooperativity parameter, *ω* (1000), and occluded binding site size on single-stranded DNA, *n*_*ss*_ (7 nucleotides) for 1–295 and ΔPR201 are the same as those reported for gp32 and *I.[11–13, 43] As in the previous section, the value of Δ*x* was taken directly from the stretching curves for dsDNA and ssDNA in the presence of protein. The salt dependent value of $F_{m}^{0}$ is from the work of Wenner *et al*.[44] The only fitting parameter varied for the best fit is the binding constant to ssDNA (*K*_*ss*_). Fits of Eq 6 are shown in Fig 10, and the results for *K*_*ss*_ are presented in Table 2. *K*_*ss*_ values for 1–295 and ΔPR201 are both about 1.0 x 10^5^ M^-1^, ~ 3 times greater than *K*_*ss*_ for gp32 and ~15 times smaller than *K*_*ss*_ for *I in 0.1 M Na^+^. Again, we conclude that the negatively charged amino acid residues near the end of CTD play an important, inhibitory role in binding of gp32 to ssDNA.

### Cross-linking of exogenous C-domain to the core domain

Additional evidence for an interaction between the C- and core domains is provided by demonstration of cross-linking between these two portions of the protein. The effects of single-stranded oligonucleotides and ionic strength on the extent of cross-linking correlate with a functional role of the C-domain in controlling nucleic acid binding. We chose the zero-length heterobifunctional cross-linker, 1-ethyl-3-[3-dimethylaminopropyl] carbodiimide (EDC), coupledwith N-hydroxysuccinimide (NHS). EDC is a powerful coupling agent of carboxylic acid groups (in abundance in the Asp-rich C-domain). Although in principle it will also react with amines (lysines on core domain) the efficiency of this reaction is significantly increased by the addition of NHS.[39]

**Fig 11:**
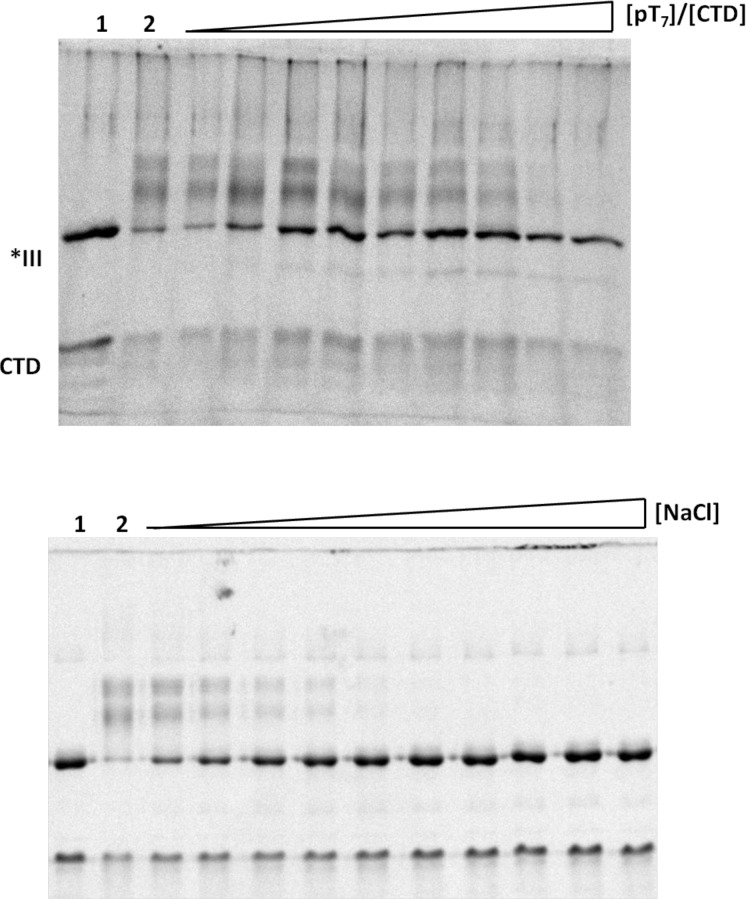
Dependence of cross-linking on oligonucleotide and salt concentration. Top panel: Effect of increasing p(dT)_7_ on cross-linking. In lane 1,*III and the CTD in lane were not cross-linked, in lane 2 they were subjected to cross-linking. In the subsequent lanes, increasing amounts of p(dT)_7_ were added so that the [p(dT)_7_/]CTD] were 0.05, 0.1, 0.15, 0.2, 0.3, 0.4, 0.5, 0.67, 1. Bottom panel: Cross-linking in the presence of increasing salt concentrations. In lane 1,*III and the CTD in lane were not cross-linked, in lane 2 they were subjected to cross-linking, and in the subsequent 10 lanes NaCl was added to the following concentrations (left to right, in mM): 20, 30, 40, 50, 72.5, 85, 100, 130, 150.

The cross-linking reaction between core and C-domain was optimized by variation of the ratio of the two proteins and the relative concentrations of EDC and NHS (see Materials and Methods for details). Typical cross-linking results under the optimized conditions, 5:1 C-domain:core domain, 0.5 mM EDC, 1.25 mM NHS) are shown in Fig 11. On SDS-PAGE, we consistently observed the formation of two relatively broad major bands of apparent molecular weights higher than core domain, along with the depletion/near disappearance of the unreacted proteins. MALDI mass-spectroscopic analysis of the cross-linked reaction mixture showed a minor band of molecular weight 36,138Da, corresponding to a 1:1 complex.

Is the C-domain cross-linked at or near the ssDNA binding site on the core domain, as posited in our model? Evidence that this is the case is provided by the effect of increasing concentrations of oligonucleotide (p(dT)_7_) on the cross-linking reaction (Fig 11, top). Approximately 50% Inhibition of the cross-linking reaction occurs at a [p(dT)_7_]:[core domain] of 1, and higher ratios effectively prevent cross-linking. Additional evidence for the relevance of cross-linking is the effect of NaCl on the reaction, which mirrors the predicted salt dependence of the closed → open conformational change model (Fig 11, bottom). Addition of 30 mMNaCl brings about a ~50% inhibition, with no evidence of cross-linked complexes seen at or above 100 mM salt.

## Discussion

In the report, we have provided a variety of evidence for a conformational change involving the C-terminal domain of gene 32 protein when it binds to single-stranded DNA. Under low salt (“strong”) binding conditions, where the rate determining step of the association kinetics is the (initial) formation of a non-cooperatively bound protein-DNA complex, the removal of the C-domain greatly alters the salt dependence of the kinetics. The positive slope plot of the log *k vs*. log [NaCl] in Fig 4 for full-length gp32 is very similar to that of *II, which only lacks the N-domain (needed for cooperativity, and not a factor at strong binding conditions). As we have noted, we attribute the positive slope to the uptake of 3 Na^+^ ions when the C-domain is released from its binding site on the core domain (*cf*. Fig 1). The same result would apply to the *II truncate. However, when the C-domain is removed, to form either *I, or *III (which also lacks the N-domain), the plots become nearly horizontal (and similar to each other), consistent with the absence of Na^+^ uptake (or release) associated with the C-domain.

The activation parameters obtained at 0.02 M NaCl show a significant dependence on the presence of the C-domain. The variation in rates among the various forms of the protein cannot be attributed to differences in ΔH^‡^, which are all around 15–25 kJ mol^-1^. However, the ΔS^‡^ values, which are all negative, are considerably more negative for the forms that possess the C-domain. Thus, in all cases the transition state is more ordered than the reactants, and the presence of the C-domain (in whole protein and *II) very much increases this difference. One possible basis for this effect is that at low salt, the C-domain, while being predominantly in the “closed” conformation (Fig 1B), is kinetically labile, rapidly opening and closing. In order for the protein to bind ssDNA, the protein must adopt exclusively an open conformation in its transition state. This results in particularly negative ΔS^‡^ values for whole protein (-100 J K^-1^ mol^-1^) and *II (-60 J K^-1^ mol^-1^).

As we have noted, the association rate constants for *II are approximations, since the linear rate dependence of concentration for this truncate does not yield a zero or near-zero y-intercept, *cf*. Fig 3). However, with this caveat, the significantly more negative ΔS^‡^ value for whole protein relative to *II (whole protein minus the N-domain) suggests that the presence of this domain might be responsible for the difference. Residues 3–6 within the N-domain, Lys-Arg-Lys-Ser-Thr (the “*LAST*” Motif), are virtually identical to residues 110–114 within the core domain, Lys-Arg-Lys-Thr-Ser.[45, 46] Conceivably, in its unbound state, the whole protein might transiently adopt an additional conformation where the C-domain interacts with virtually the same (*LAST*) residues in the N-domain as it does when it binds to the core domain LAST residues, forming the closed, ssDNA-binding conformer. This additional conformation would lead to a higher net entropy for the unbound protein reactant, and therefore a more negative ΔS^‡^ (the entropy of the transition state remaining essentially unchanged).

These results may reflect a more general regulatory mechanism for DNA binding. A similar mechanism was reported for the single-stranded DNA binding protein from T7, gp2.5.[24, 47]Both proteins T4 gp32 and T7 gp2.5 have an acidic C-terminus that regulates their DNA binding. T7 gp2.5 lacks DNA binding cooperativity, a significant difference from gp32. T7 gp2.5 also has a weaker salt dependence to its DNA binding, indicating a weaker electrostatic interaction between its C-terminus and its DNA binding site.[48] The results presented here support a general mechanism of electrostatic regulation for DNA binding of SSBs presented in earlier studies of T7 gp2.5.

Although there has been a suggestion that the C-domain of gene 32 protein can form an ordered α-helical bundle,[14] there is no experimental evidence for any higher-order structure in this domain. In this regard, the C-domain of each *E*.*coli* tetrameric SSB polypeptide consists of an intrinsically disordered 56-residue region with an acidic 9 amino acid terminus.[49, 50] As in the case of gp32, the *E*. coli SSB C-domain is the site of interaction with a large number of proteins that function in replication, recombination, and repair.[51]

The presence of an unstructured, acidic C-domain participating in a large number of heterotypic protein-protein interactions essential to DNA metabolism may be a property shared by many SSBs, including the C-domains of the *E*. *coli* SSB and the T7 gene 2.5 protein[52]. With the requirement of recognizing many protein partners, it would seem that structural flexibility in this part of the protein would optimize selectivity and kinetically facilitate transfer from one heterotypic interaction to another. This also applies to the closed ⇌ open conformational change in gp32.

External and internal protein-protein interactions are likely to involve (different) specific locations on the C-domain. This is well illustrated by the ΔPR201 variant of gp32, where we show that the loss of residues 292–296 in the C-domain significantly alters the ssDNA association kinetics and dsDNA helix-destabilization properties of this protein. A full understanding of the roles of the C-domain of gp32 and other SSBs in DNA metabolism will require delineation of other critical interactive and regulatory sites in these flexible, unstructured regions.
